# Strengthening Equitable Access to Care and Support for Children with Cerebral Palsy and Their Caregivers

**DOI:** 10.3390/children10060994

**Published:** 2023-06-01

**Authors:** Aysha Jawed, Michelle Mowry

**Affiliations:** 1 Johns Hopkins Children’s Center, Baltimore, MD 21287, USA; mgippri1@jhmi.edu; 2 Department of Pediatric Social Work, Johns Hopkins Children’s Center, Baltimore, MD 21287, USA; 3 Department of Pediatric Nursing, Johns Hopkins Children’s Center, Baltimore, MD 21287, USA

**Keywords:** cerebral palsy, pediatric, health equity, caregiver preference, family-centered care, risk and resilience, biopsychosocial

## Abstract

Cerebral palsy is one of the most prevalent groups of motor disorders affecting children and adults across the world. As increasingly more children with cerebral palsy are living longer into adulthood, it is ever more crucial to ensure access to timely and needed early intervention from the onset of diagnosis, on a continuum, to optimize medical, developmental, socio-emotional, and academic outcomes for these children over time. The American Academy of Pediatrics (AAP), in collaboration with the American Academy of Cerebral Palsy and Developmental Medicine (AACPDM), substantially revised the clinical practice guidelines for cerebral palsy in 2022, after their prior publication of the guidelines in 2006. The revised guidelines account for a range of considerations that are in line with the biopsychosocial, risk and resilience, and family-centered care models as well as promote a more strengths-based approach to care. Furthermore, there is increased emphasis in the guidelines on promoting equitable access to care as part of contributing towards health equity for all children with cerebral palsy. In addition, the 2022 guidelines clearly present recommendations for earlier diagnosis of cerebral palsy, potentially as early as infancy, as the basis for activating access to early intervention services for children that can bolster their neuroplasticity and global development from an earlier age onward. We consolidate the existing literature on caregiver perceptions, beliefs and concerns surrounding earlier diagnosis of cerebral palsy and connect them to the recommendations in the revised guidelines. We also delineate several considerations surrounding education for healthcare providers and caregivers of children in navigating the chronicity of cerebral palsy in both community and healthcare contexts. There is a scant amount of literature on cerebral palsy across traditional and nontraditional sources of media in published studies, which we also review. Lastly, we present a wealth of recommendations for further research and practice that account for the revised 2022 guidelines, caregiver preferences and acceptability of care, and health equity as the bases for strengthening equitable access to care for children with cerebral palsy on a continuum as they transition into adulthood.

## 1. Introduction

Cerebral palsy is the most prevalent group of motor disorders of the nervous system among children, which affects 1 to 4 out of 1000 children annually [1]. Approximately 8000 neonates and infants, as well as 1200 to 1500 school-aged children, are diagnosed with cerebral palsy each year in the U.S. [2]. Cerebral palsy can also create or increase the risk for multiple medical complications and conditions among children, including epilepsy, speech and eating difficulties, respiratory disorders, spasticity, gastrointestinal and nutritional disorders, cognitive, perception and communication issues, chronic pain and many more such complications [3,4,5,6,7]. Notably, spastic cerebral palsy affects up to 80% of children with cerebral palsy [1]. In addition, fewer than 5% of suspected cases of cerebral palsy are confirmed for diagnosis prior to 5 years of age among children [8].

In addition, a child with cerebral palsy may experience significant global developmental delays and significant challenges with meeting developmental milestones [9,10,11]. Similar to many developmental disorders, cerebral palsy can range on a spectrum of severity, with respect to functioning, that can encompass ambulation, use of assisted devices and significant activity limitations (e.g., wheelchair-bound) [12].

Based on the newly revised clinical practice guidelines for cerebral palsy authored by the American Academy of Pediatrics (AAP) in collaboration with the American Academy for Cerebral Palsy and Developmental Medicine (AACPDM), there have been several evidence-based risk factors for cerebral palsy uncovered that can present both prenatally and postnatally for children. Several of these risk factors have consistently manifested at an earlier age among children, beginning during infancy. These risk factors include prematurity, perinatal difficulties, motor asymmetry (e.g., early hand preference), inability to sit independently by 9 months of age, and poor muscle tone [13]. For these reasons, the revised guidelines account for earlier diagnosis as the path for addressing global developmental delays and generating neuroplasticity from the onset of diagnosis [13]. However, it is important to note that risk does not equate to disability, and there are different spectrums of functional outcomes for patients with cerebral palsy with respect to skills in gross and fine motor capabilities, communication, feeding, vision, and activities of daily living, which all impact functioning, based on the 5 F framework and the International Classification of Functioning, Disability, and Health [14,15].

Notably, there are certainly mixed opinions in the healthcare community about making a diagnosis of cerebral palsy earlier in the child’s life during instances where it may not be the case and especially in light of the fact that this group of motor disorders is one of exclusion [8,16]. It follows that healthcare providers oftentimes take a more precautionary approach to diagnosis (e.g., delay in imaging with concern for radiation exposure given the younger age of the child), which may not always be in line with the goals, values, concerns and priorities of the child’s family, thereby making it harder for shared decision-making between the child’s caregivers and healthcare team.

Of note, the clinical practice guidelines on cerebral palsy published in 2022 by the AAP and AACPDM have been substantially revised since the prior edition in 2006. The newly revised guidelines also account for a range of considerations that encompass constructs across the biopsychosocial, risk and resilience, and family-centered care frameworks. For example, the 2022 guidelines build on the strengths of a child’s family, account for health equity considerations, further support early diagnosis of cerebral palsy as the basis to increase access to timely and needed services earlier in the child’s life as a predictor of optimal future outcomes and heighten the visibility and accountability of the primary medical home and specialty care in supporting the child and family across the chronic trajectory of cerebral palsy [13]. These guidelines have further extended the 2006 guidelines and are much more up-to-date in the context of our current times, as increasingly more children are living longer with developmental disabilities, which further makes it imperative to ensure access to equitable care on a continuum for these children as they transition into adulthood.

We will review the key differences between the 2016 and 2022 AAP and AACPDM cerebral palsy guidelines. We will also synthesize the primary findings across published studies on caregiver beliefs, perceptions and concerns surrounding earlier and later diagnoses of cerebral palsy for their children as the basis to tie-in the consistency in findings as part of the revisions in the guidelines. The scant coverage of cerebral palsy in published studies, considering both traditional and nontraditional sources of media, will be presented, along with recommendations for further research and practice which account for the revised guidelines, caregiver preferences and acceptability of care, and media coverage of cerebral palsy to heighten knowledge and awareness across the world as the bases to mobilize further advocacy and resources in assuring equitable delivery of care for these patients.

## 2. Key Differences between the 2006 and 2022 AAP and AACPDM Cerebral Palsy Guidelines

There has been increased focus on managed care over the past 16 years, and further focus on patient-and-family-centered care considerations, along with increased recognition of the chronicity of developmental disabilities over the past couple of decades, especially as more individuals are living longer with disabilities in light of advances in science, technology and medicine. Further, there is increased renewed focus on the pediatric medical home for children with complex medical needs and optimizing global development from the onset of diagnosis. More research has also been conducted on predictors, determinants and treatment considerations for cerebral palsy over the years to uncover evidence-based indicators as targets for intervention in supporting children with cerebral palsy and their families. Additionally, in the context of our digital era during these contemporary times, it is increasingly more crucial to mitigate the rising trends of misinformation and disinformation across all communication mediums that could adversely impact the care of patients with cerebral palsy. In addition, in light of the U.S. government’s initiative “Healthy People 2030”, which accounts for prioritization and continued work in health equity, there is an even greater emphasis in our current era on bridging the divide in access to care for patients with and without disabilities.

In the 2022 AAP and AACPDM cerebral palsy guidelines, there is significant focus on early identification as the basis for securing timely and needed intervention following diagnosis of cerebral palsy among children. This recommendation is built on the fact that, over time, for a range of reasons, there have been multiple delays in diagnosing cerebral palsy, affecting many children, given that it is a diagnosis of exclusion. From safety, liability and ethical perspectives, pediatricians and developmental specialists have been more conservative and cautious in providing this diagnosis of exclusion. There are also varying degrees of sensitivity and specificity among the different diagnostic tests for cerebral palsy. However, there are tests that have demonstrated high sensitivity and specificity in diagnosis, which are accounted for in the 2022 guidelines. Table 1 presents the key takeaways from the revised guidelines as well as ethical resources in guiding diagnosis of cerebral palsy. For infants older than 5 months of age, specific scores on the Hammersmith Infant Neurological Examination (less than 73), in combination with abnormal findings from magnetic resonance imaging (MRI) in regions of the brain responsible for regulating motor skills, have consistently demonstrated an accurate diagnosis of cerebral palsy 90% of the time for infants at 6, 9 and 12 months of age [17]. Among infants younger than 5 months of age, the Prechtl’s General Movements Assessment, in combination with abnormal MRI findings in cerebral regions pertaining to motor skills, have consistently yielded an accurate diagnosis of cerebral palsy 95% of the time for these infants [17]. These tests, in combination with imaging, could yield promise in facilitating earlier diagnosis of cerebral palsy as early as infancy. Additional tests have demonstrated low-to-moderate degrees of precision in diagnosis for cerebral palsy (e.g., Test of Infant Motor Performance, and the Developmental Assessment of Youth and Children) [17].

The 2022 AAP and AACPDM guidelines strongly encourage pediatricians to initiate this comprehensive diagnostic neuromotor work-up of children, especially with respect to motor milestones and muscle tone, along with potential brain imaging. The guidelines recommend facilitating this work-up from the onset of any symptoms in line with cerebral palsy by referring to a specialist to complete this work-up sooner rather than later, which will also account for the long waiting lists in securing appointments with specialists [13]. These guidelines also recommend that pediatricians connect children with suspected or confirmed cerebral palsy with early intervention programs for the initiation of evidence-based developmental therapies that could potentially increase neuroplasticity in children during this critical time when the greatest gains are possible [17]. Based on a systematic review, neuroplasticity improved among patients with cerebral palsy through motor and speech interventions; however, further work is warranted to assess for consistency in these findings across studies and programs that implement the principles of motor learning as a predictor of improved neuroplasticity [18]. Furthermore, earlier access to these therapies could improve medical and developmental outcomes for these children in the future, which in turn could optimize their adaptability to change, activities of daily living and quality of life [17].

There is also increased emphasis in the 2022 AAP and AACPDM guidelines on addressing pain and discomfort among children with cerebral palsy from the onset, and on a continuum, by identifying sources of pain and a plan to alleviate them [13]. Furthermore, the guidelines also delineate treating each child’s new onset of symptoms or functional decline as the chief focus of presenting medical complications rather than initially attributing these symptoms to underlying cerebral palsy without any further investigation for sources of symptoms. These guidelines also recommend involvement from the palliative care team in assisting these children to achieve pain and symptom management, as well as minimize discomfort. In addition, the guidelines also suggest initiation in transition of care for children with cerebral palsy during their early preteen and adolescent years (between 12 to 14 years of age) to begin preparing them for the coming transition several years later into late adolescence and early adulthood. In the prior guidelines, there was no recommended time range for initiation of discussions on transition of care after 12 years of age. The 2022 AAP and AACPDM guidelines also recommend comprehensive written physician sign-out as the child transitions from pediatric to adult care.

Overall, the 2022 AAP and AACPDM guidelines advocate for systemic change, across both community and inpatient contexts, for strengthening equity, affordability and accessibility of healthcare services and resources for children with cerebral palsy as the bases for optimizing their long-term future in integrating into society, acquiring life-skills and meeting their developmental, socio-emotional and academic needs over time. The guidelines also seek to bridge the divide in access to timely and needed care for these children and their families in ways that transcend all racial, ethnic, age, and socioeconomic groups, thereby seeking to heighten health equity and address social determinants of health in the care of children with cerebral palsy. These guidelines are also much more in line with promoting components of patient-and-family-centered care, the risk and resilience model, and a strengths-based approach with their increased focus on the strengths as well as both the intrinsic and extrinsic resources of the child’s family, decision-making by the family, and collaboration among the family and the child’s multidisciplinary care team as one united team for the child. In the prior 2006 AAP and AACPDM guidelines, assessment of family needs as the basis for connecting them with resources was the primary method used to evaluate the family’s resources in meeting the needs of the child and there was not much focus on identifying strengths of the family as an essential predictor in navigating care and management of cerebral palsy. In the 2022 AAP and AACPDM guidelines, there is also substantial focus on immersing the pediatrician in community contexts by increasing communication and collaboration with the child’s resource providers, including therapists, schools, inpatient providers (e.g., complex care team), community play and support groups, and sources of financial resources, among others. In addition, the 2022 guidelines clearly present that children with disabilities and specialized healthcare needs inclusive of cerebral palsy are also at increased risk of child maltreatment. In turn, recommendations in the guidelines support pediatricians’ efforts to take an active role in educating caregivers of these children on indicators and risk factors for child maltreatment as well as connecting them with resources in cases where there is potential risk. In the prior guidelines, healthcare providers were encouraged to make referrals to the range of these services, but there was no emphasis on engaging in direct communication with the families of children with cerebral palsy on the availability of these community resources.

In addition, based on research conducted since the prior guidelines, cerebral palsy has been successfully diagnosed in children as young as 9 months of age. It follows that the 2022 AAP and AACPDM guidelines recommend that screening for developmental disabilities inclusive of cerebral palsy begin for infants at the age of 9 months and continue, as they grow into toddlers, at both 18 months and 30 months of age irrespective of known risk factors. These guidelines promote, for children with cerebral palsy, routine well-child visits with primary care that include receiving vaccinations on-schedule throughout their childhood and adolescent years. Furthermore, there is both increased and renewed emphasis on the multidisciplinary village of providers involved in addressing, on a continuum, the medical, developmental, socio-emotional, academic and further needs of children with cerebral palsy. For example, pediatricians could potentially work closely with the child’s teachers, developmental therapists, and social workers in the community to prepare a comprehensive individualized educational plan that seeks to increase adaptability, functioning, and social skills for a child with cerebral palsy both inside and outside the classroom. The 2022 AAP and AACPDM guidelines also further propose, in the care of these children, taking a more biopsychosocial approach rather than a biomedical approach with their caregivers by viewing the pediatrician as a catalyst in encouraging caregivers to engage in different social, recreational and other kinds of activities and groups in the community that are in line with the child’s interests and development. In addition, the guidelines also encourage pediatricians to further provide recommendations for recreation and athletics that account for access to adaptive equipment whenever indicated.

## 3. Caregiver Support of Early Diagnosis for Cerebral Palsy in Children

Early diagnosis of cerebral palsy among children is also a predictor of support for their caregivers [9,16,19,20,21,22]. In one study, caregivers have verbalized that early diagnosis has improved their understanding of the complex and specialized needs of their child [19]. In another study, early diagnosis has also empowered caregivers to feel a sense of control following revelation of their child’s diagnosis [22]. A different study uncovered that caregivers prioritized connecting their child and family to early intervention services at the onset of diagnosis [16]. Also, in one study, increased satisfaction in care and navigating the chronicity of cerebral palsy was ranked higher among caregivers who had learned about the importance of an earlier diagnosis of cerebral palsy for their children [21].

In a study among 67% of children who were diagnosed with cerebral palsy before 2 years of age, nearly 40% of caregivers expressed that the diagnosis was a little too late or very delayed [23]. In two studies, caregivers reported dissatisfaction with the timing of diagnosis for cerebral palsy among their children [24,25]. Furthermore in one of these studies, nearly 41% of mothers reported that the diagnosis was ultimately delayed for their child [25].

In another study, parents reported that they felt blindsided after receiving a diagnosis of cerebral palsy for their child [9]. Furthermore, in this study, parents also described feeling poorly-equipped to meet the complex and specialized medical needs of their child. They also felt that they were ill-prepared from the onset of diagnosis, given the limited information and resources provided to them at that time. In the same study, caregivers also described feeling that their concerns were minimized or dismissed by healthcare providers and further shared reports of poor communication surrounding the process of diagnosis as well as signs, symptoms, and indicators to monitor over time. For several of these caregivers, a delay in diagnosis and limited information about the implications and trajectory of it ultimately resulted in a delay in accessing early intervention services that could potentially have helped address developmental milestones and neuroplasticity for the child from time of infancy. In some of these cases, caregivers experienced immense guilt stemming from feeling that they had not advocated enough for their child to receive the diagnosis or felt that they contributed or caused the diagnosis or delay in diagnosis from not having any knowledge and awareness of its symptomatology, which they attributed to unclear communication with the child’s healthcare providers.

## 4. Education for Healthcare Providers and Caregivers on Early Diagnosis of Cerebral Palsy

The newly revised 2022 AAP and AACPDM guidelines emphasize the importance of early diagnosis of cerebral palsy. Further education for healthcare providers involved in the care of children could in turn focus on identifying risk factors for cerebral palsy (e.g., not meeting developmental milestones, gross motor delays, etc.) from time of routine well-child visits during infancy and onward. As mentioned above, cerebral palsy can be diagnosed in infants from as early as 9 months of age. It follows that continued assessment for any possible symptoms which could be in line with the manifestation of cerebral palsy should occur at developmental intervals, and therefore at 9 months, 18 months and 30 months of age, as well in between well-child visits to decrease the chance of missing any potential risk factors that could indicate concern for suspected cerebral palsy. Furthermore, education for healthcare providers on the sequelae of cerebral palsy associated with a range of other developmental and medical disorders (e.g., epilepsy and respiratory conditions) is pivotal in addressing the constellation of medical and developmental complexities from the onset that could arise for the child, which in turn could assure timely access to much-needed resources to support the child and their family. In addition, strengthening education for healthcare providers could potentially have a trickle-downstream impact on heightening caregiver education on a range of dimensions for navigating the trajectory of cerebral palsy with their child, which subsequently could increase support and mitigate apprehension, suffering and anxiety among the caregivers.

## 5. Prevalent Typologies of Diagnostic Imaging for Cerebral Palsy

Several studies have uncovered recommendations of typologies of diagnostic imaging for cerebral palsy. In one study, findings revealed that magnetoencephalography was promising for uncovering processing of abnormal neural information, and recommended future work in this domain [26]. In another study, early diagnosis of cerebral palsy among very-preterm infants involved utilization of sensorimotor-tract biomarkers in assessing for any presence of brain damage among these infants [27]. One study uncovered increased accuracy in the detection of cerebral palsy from MRI imaging of segmented brain tissue based on the 3D model of a convolutional neural network [28]. A different study revealed that fractional anisotropy in the corticospinal tract at the internal capsule level yielded efficacy in identifying infants with periventricular white-matter injury as having or not having spastic cerebral palsy [29]. These findings were furthermore strongly correlated with motor-function scores of infants in the study. Table 2 presents a breakdown of the typologies of imaging utilized, in published studies, for detection of cerebral palsy.

## 6. Increased Presence of Healthcare Providers in the Community Care of Children with Cerebral Palsy

Findings from one study elucidated that caregivers from all walks of life felt challenged in navigating the complexities of services within the community required to connect their child with early-intervention programs from the onset of diagnosis [9]. The newly revised 2022 AAP and AACPDM guidelines recommend that healthcare providers could be more actively involved in this process for these children and their families. It follows that, by healthcare providers taking on a more visible presence and active role in referring children with cerebral palsy to community programs for early intervention, caregivers could subsequently access timely and needed care for their child, which in turn could contribute towards mitigating any apprehension with navigating the healthcare, educational, developmental and other complex systems.

## 7. Existing State of Coverage on Cerebral Palsy across Published Studies and Social Media

Parents in one study reported that the internet presented more information about the symptomatology of cerebral palsy and, in turn, heightened their knowledge and awareness of the condition much more than the content shared by their child’s healthcare providers [9]. In this study, the majority of the parents felt that online support groups were the primary agent in supporting them in more quickly connecting with community resources for their child. Notably, there have been only two published studies conducted on assessing content posted on social media platforms [30,31]. In one of these studies, involving a content analysis of YouTube videos solely in Brazilian-Portuguese, 90 videos were examined for content across diverse sources and deliverers of information centered on several domains surrounding cerebral palsy [30]. These domains included the following: (1) multidisciplinary treatment; (2) etiology and prevention; (3) classification, characteristics and prognosis; (4) clinical aspects and comorbidities; and (5) functional aspects. Nearly 70% of these videos emphasized multidisciplinary treatment, through lectures and interviews that involved options for neurodevelopmental treatment, bimanual hand and arm training, intensive therapies and orthopedic surgeries.

Another study involved critical examination of selective dorsal rhizotomy, as a surgical intervention to alleviate spasticity of cerebral palsy, among children across three prevalent social media platforms: Facebook, YouTube, and Twitter [31]. In this study, communication and content about this surgical intervention was reviewed across 185 Facebook groups, 97 YouTube videos, and 14 Twitter accounts. Communication and content revealed themes pertaining to guidance and challenges in navigating everyday life and inequities pertaining to access of timely and needed resources, success in meeting developmental milestones and optimizing quality of life, as well as emotional support for caregivers as discussions surrounding surgery also extended to navigating the chronic trajectory of their child’s cerebral palsy on a daily basis. In addition, this study also uncovered that caregivers identified social media as a source of a second opinion informing their decision about pursuing selective dorsal rhizotomy for their child, in order to alleviate spasticity.

In a different study, one that examined coverage of twelve campaigns pertaining to developmental disorders, one campaign (Día Mundial de la Parálisis Cerebral) centered on cerebral palsy celebration and awareness [32]. There was an increased uptake of searches for this campaign on Google and a wider reach on Twitter. Among the campaigns examined, this campaign yielded the top ten hashtags across campaign trends consistently visible on Twitter during their pendency. Currently on Facebook, the online support groups that consist of the greatest number of members are the Cerebral Palsy Support Group (~29,000 members) and the Cerebral Palsy PARENTS information group (~20,000 members). Both groups are closed and private online support networks intended to promote communication exchange among caregivers of children with cerebral palsy. At this time on Instagram, the pages that have the greatest number of followers are the Cerebral Palsy Foundation (~26,000 followers), followed by the Cerebral Palsy Alliance (~12,000 followers).

Increased social media coverage yields promise in heightening knowledge and awareness on a range of considerations for cerebral palsy across the world, given the global reach of social media during this digital time. It follows that this social media coverage could also serve as a cue to action and in turn mobilize advocacy and resources in working towards equitable access to care for these patients. As social media rises as a health communication medium, it is ever more critical to identify how it impacts patients with cerebral palsy and their support networks as the foundation for designing comprehensive interventions that account for social media as a practice setting, especially for uninsured and underinsured patients, at least in the meantime, until they have access to developmental services.

## 8. Limitations of Clinical Practice Guidelines and Content Coverage for Cerebral Palsy

One of the central limitations of the 2022 AAP and AACPDM guidelines pertains to their clinical nature, which precludes addressing operational considerations related to staffing and financing of services as part of increasing sustainability, affordability and access for patients with cerebral palsy and their families. The 2022 revised guidelines strongly support early diagnosis as an integral pathway to mobilizing resources and navigating the chronic trajectory of cerebral palsy, which includes accounting for emerging symptoms as targets for intervention in optimizing quality of life and global development from the onset of diagnosis. It follows that, without a mechanism of payment and increased multidisciplinary staffing to meet the complex needs of these patients, assuring follow-through with these guidelines could be challenging in light of the staffing shortage crisis and resource limitations we are currently experiencing in our healthcare climate, both in the USA and across the world. One recommendation for future revisions to these guidelines is to account for operational (e.g., financial and staffing), funding, and community-engagement considerations in assuring continued access to timely and needed services for patients with cerebral palsy from the onset of diagnosis as a measure of sustainability in the chronic care of these patients. Involvement from institutional stakeholders, government and healthcare administrators could yield more suggestions for realistically addressing the feasibility of implementing these practice guidelines in the context of these logistical considerations. To date, there have been no published studies that have examined the impact of social media coverage on operational considerations and support from the government, for-profit, and non-profit organizations in navigating cerebral palsy. It follows that future work could assess coverage of the revised 2022 guidelines as the basis for uncovering any content on challenges with implementing and scaling these guidelines, given the limitations in both community and healthcare infrastructure.

## 9. Clinical and Research Implications

We thoroughly reviewed the revised 2022 AAP and AACPDM guidelines for cerebral palsy and critically assessed differences between them and the former iteration of the guidelines published in 2006. Reviewing the differences helped uncover a more comprehensive understanding of chronic care considerations surrounding cerebral palsy that take into account advances in biomedical research, the biopsychosocial model, social determinants of health, risk and resilience, and family-centered care in meeting the complex and specialized needs of these patients.

Over the past 16 years, a multitude of risk factors and practice considerations (e.g., early diagnosis and intervention) have consistently been demonstrated to be predictors for optimizing quality of life and better developmental outcomes and transitions from pediatric to adult care for patients with cerebral palsy. Based on the consistency in these findings, which ultimately formed the heart of the revised guidelines, it is crucial to continue research on these determinants and clinical factors, as well as emerging ones, to continue staying up-to-date on best practices in the care of patients with cerebral palsy across the developmental cycle.

In addition, it is imperative to account for the reality that, with trending misinformation, there is the implication that non-credible content could deemphasize the child’s strengths. All children with cerebral palsy have the potential to learn, and it is crucial that content coverage across both traditional and nontraditional health communication mediums accounts for the strengths-based perspective and community contexts elucidated in the revised 2022 guidelines, which promote participation in education as well as after-school and community activities. Of note, given the complexity of children with gross motor, manual, and communication function of GMFCS V, MACS V and CFCS IV/V, the healthcare system oftentimes only interfaces with the severest functional outcomes of early childhood disability, with multiple co-morbidities that include seizures, dysphasia, visual disability, growth failure and dislocated hips. There is a much more expanded range of trajectories that, except in extreme cases, cannot be predicted by neuroimaging.

## 10. Recommendations for Future Research and Practice

The revisions in the AAP and AACPDM cerebral palsy guidelines between 2006 to 2022 reflect substantial changes over the past sixteen years. One of the key differences between iterations of the guidelines is the increased focus on early diagnosis as the basis for activating early intervention in promoting optimal developmental and medical outcomes, prognosis and quality of life for infants with cerebral palsy, potentially from the time of infancy. Another key difference is having the healthcare provider take on an active role as a broker in supporting families in mobilizing a range of healthcare services and resources to meet the medical, developmental, socio-emotional, and academic needs of children and strengthen support for their families, as they navigate the chronicity of cerebral palsy for their children. It follows that increased education for healthcare providers on evidence-based recommendations to screen for motor delays that could be attributed to cerebral palsy could heighten early diagnosis whenever indicated. Based on the revised 2022 AAP and AACPDM guidelines, assessing for cerebral palsy at 9 months, 18 months and 30 months of age (as well as in-between as needed) could also optimize early diagnosis for the activation of timely and needed care of children. Further, in line with consistent findings across multiple studies, increased early diagnosis of cerebral palsy in children is associated with stronger support, resilience and satisfaction (in care) for their caregivers in navigating the complexities and chronicity of this developmental disorder.

In addition, there is also increased focus on initiating transition of care within the 12 to 14 years of age window of time. It follows that increased education for healthcare providers on initiating these discussions and identifying equivalent adult care providers (e.g., adult primary care providers, neurologists and developmental therapists) during this window of time for children could be a predictor of sustained care and management of their cerebral palsy as they transition into adulthood. Furthermore, given the efficacy of enablement counseling for patients in all stages of life with cerebral palsy, it could be immensely helpful in integrating this practice as part of the continuity of care for these patients throughout their lifespan [15,33,34,35].

Also, in the revised 2022 guidelines, there is an increased focus on addressing sources of pain among children with cerebral palsy that involves not attributing pain solely to the child’s underlying cerebral palsy without investigating any potential organic or medical causal factors for the pain. Future efforts in education for healthcare providers can also reinforce a multimodal assessment for the totality of possible sources of pain, given that the presentation of pain could be a symptom of another problem, one which could be much harder for children to express, especially among children who are non-verbal.

In addition, there has been scant coverage of content on cerebral palsy across social media in the existing published literature. In the context of our digital era, during contemporary times, social media has taken a much more increased and visible presence as an influencer and bearer of information across many domains of life [36]. Notably, in two studies investigated, social media and campaign coverage of cerebral palsy were in the Portuguese-Brazilian or Spanish languages and meant primarily to reach the national populations of Brazil and Spain [30,32]. Future research could involve critically examining social media content in English and additional languages to account for coverage of content that could be relevant to more segments of the global population of children with cerebral palsy and their caregivers. In addition, considering the leading online support groups on Facebook, each one is a closed and private group. Consequently, it was not possible to examine the communication among members of the groups without actually being a member of each of these groups. Future directions of research could entail healthcare providers taking a more active role on social media by potentially joining these groups to engage in the discourse, present credible recommendations on community resources and therapies, and mitigate any misinformation and disinformation that is not in line with the evidence-based recommendations presented in the newly revised 2022 AAP and AACPDM guidelines. In addition, conducting a content analysis to identify patterns, themes and trends emerging in the communication within these groups and the popular pages on cerebral palsy across Instagram could yield immense value in understanding the impact of cerebral palsy on caregivers, as well as what has and has not worked for them, through understanding their narratives and perspectives, which in turn could contribute towards developing interventions to support this caregiver population as they navigate the chronicity of their child’s cerebral palsy.

## 11. Conclusions

Taking everything into consideration, as more children with cerebral palsy are living longer into adulthood, it is ever more crucial to find ways that will strengthen support for this population and their caregivers, who are instrumental in helping them navigate the first years of their lives as they transition into adulthood. Accounting for the recommendations in the newly revised 2022 AAP and AACPDM guidelines in practice could be a predictor of increasing equitable access to a range of resources and services that could more optimally meet the complex and specialized medical and developmental needs of these children. Furthermore, one premise of these revised guidelines also yields more potential directions for continued clinical research, specifically, developing and implementing interventions, as the basis for assessing their efficacy in consideration of their inclusion in evidence-based practice among children with cerebral palsy. These guidelines, along with future work in continuing to assess and expand these guidelines over time to support this patient population, will also contribute towards the larger goals of the World Health Organization (WHO), the United Nations International Children’s Emergency Fund (UNICEF), and “Healthy People 2030” in optimizing equitable access to care for individuals with disabilities on both global and national levels.

## Figures and Tables

**Table 1:** AAP and AADMCP 2022 key guidelines and ethical resources.

**AAP and AADMCP 2022 Key Guidelines**
Early identification as the basis for securing timely and needed intervention following diagnosis of cerebral palsy among children.
For infants older than 5 months of age, specific scores on the Hammersmith Infant Neurological Examination (less than 73) in combination with abnormal findings from magnetic resonance imaging (MRI) in regions of the brain responsible for regulating motor skills have consistently demonstrated an accurate diagnosis of cerebral palsy 90% of the time for infants at 6, 9 and 12 months of age.
Among infants younger than 5 months of age, the Prechtl’s General Movements Assessment in combination with abnormal MRI findings in cerebral regions pertaining to motor skills have consistently yielded an accurate diagnosis of cerebral palsy 95% of the time for these infants.
Strongly encourage pediatricians to initiate a comprehensive diagnostic neuromotor work-up of children especially with respect to motor milestones and muscle tone, along with potential brain imaging.
Facilitate this work-up from the onset of any symptoms in line with cerebral palsy by referral to a specialist to complete this work-up sooner rather than later, which will also account for the long waiting lists in securing appointments with specialists.
Support pediatricians in connecting children with suspected or confirmed cerebral palsy with early intervention programs for the initiation of evidence-based developmental therapies that could potentially increase neuroplasticity in children during this critical time when the greatest gains are possible.
Address pain and discomfort among children with cerebral palsy from the onset and on a continuum by identifying sources of pain and a plan to alleviate them.
Treat each child’s new onset of symptoms or functional decline as the chief focus of presenting medical complications rather than initially attributing these symptoms to underlying cerebral palsy without any further investigation for sources of symptoms.
Involvement from the palliative care team in assisting these children to achieve pain and symptom management as well as minimize discomfort.
Initiation in transition of care for children with cerebral palsy during their early preteen and adolescent years (between 12 to 14 years of age) to begin preparing them for the coming transition several years later into late adolescence and early adulthood.
Comprehensive written physician sign-out as the child transitions from pediatric to adult care.
Immersing the pediatrician in community contexts by increasing communication and collaboration with the child’s resource providers, including therapists, schools, inpatient providers (e.g., complex care team), community play and support groups, and sources of financial resources, among others.
Support pediatricians in taking an active role in educating caregivers of these children on indicators and risk factors for child maltreatment, as well as connecting them with resources in cases where there is potential risk of child maltreatment.
Screening for developmental disabilities inclusive of cerebral palsy beginning for infants at the age of 9 months and continuing, as they grow into toddlers, at both 18 months and 30 months of age, irrespective of known risk factors.
Promote routine well-child visits for children with cerebral palsy with primary care that include receiving vaccinations on-schedule throughout their childhood and adolescent years.
Multidisciplinary village of providers involved to address, on a continuum, the medical, developmental, socio-emotional, academic and further needs of children with cerebral palsy.
In the care of these children, take a more biopsychosocial approach rather than a biomedical approach with their caregivers by viewing the pediatrician as a catalyst in encouraging caregivers to engage in different social, recreational and other kinds of activities and groups in the community that are in line with the child’s interests and development.
Encourage pediatricians to further provide recommendations for recreation and athletics that account for access to adaptive equipment whenever indicated.
**Ethical Resources for Diagnosis Guidance**
GMFCS—https://cerebralpalsy.org.au/our-research/about-cerebral-palsy/what-is-cerebral-palsy/severity-of-cerebral-palsy/gross-motor-function-classification-system/ (accessed on 26 May 2023).
MACS—https://cerebralpalsy.org.au/our-research/about-cerebral-palsy/what-is-cerebral-palsy/severity-of-cerebral-palsy/manual-ability-classification-system/ (accessed on 26 May 2023).
CFCS—https://cerebralpalsy.org.au/our-research/about-cerebral-palsy/what-is-cerebral-palsy/severity-of-cerebral-palsy/communication-function-classification-system-cfcs/ (accessed on 26 May 2023).

**Table 2:** Typologies of imaging and key findings across studies on diagnostic imaging for cerebral palsy.

First Author, Year	Typology of Imaging	Key Findings
Wilson, 201	Magnetoencephalography	Magnetoencephalography was promising in uncovering processing of abnormal neural information.
Parikh, 2019	Sensorimotor-tract biomarkers	Early diagnosis of cerebral palsy among very-preterm infants involved utilization of sensorimotor tract biomarkers in assessing for any presence of brain damage among these infants.
Yang, 2021	MRI imaging of segmented brain tissue based on the 3D model on convolutional neural network	Increased accuracy in the detection of cerebral palsy from MRI imaging of segmented brain tissue based on the 3D model on convolutional neural network.
Jiang, 2019	Fractional anisotropy in the corticospinal tract at the internal capsule level	Fractional anisotropy in the corticospinal tract at the internal capsule level yielded efficacy in identifying infants with periventricular white matter injury as having or not having spastic cerebral palsy; these findings were furthermore strongly correlated with motor-function scores of infants in the study.

## Data Availability

No new data were created or analyzed in this study. Data sharing is not applicable to this article.
